# Supplementary material for: Health-Screening-Based Chronic Obstructive Pulmonary Disease and Its Effect on Cardiovascular Disease Risk
Source: J Clin Med. 2022 Jun 2;11(11):3181. doi: 10.3390/jcm11113181 (PMC9181412; doi:10.3390/jcm11113181)
Supplement: Supplementary file 1 [file jcm-11-03181-s001.zip › jcm-1726848-supplementary.pdf]

Supplementary Table S1. TRIPOD Checklist: Prediction Model Development and Validatio n

Section/Topic			Checklist Item	Page
**Title and abstract**				
Title	1	D;V	Identify the study as developing and/or validating a multivariable prediction model, the target population, and the outcome to be predicted.	1
Abstract	2	D;V	Provide a summary of objectives, study design, setting, participants, sample size, predictors, outcome, statistical analysis, results, and conclusions.	1
**Introduction**				
Background and objectives	3a	D;V	Explain the medical context (including whether diagnostic or prognostic) and rationale for developing or validating the multivariable prediction model, including references to existing models.	1-2
	3b	D;V	Specify the objectives, including whether the study describes the development or validation of the model or both.	1-2
**Methods**				
Source of data	4a	D;V	Describe the study design or source of data (e.g., randomized trial, cohort, or registry data), separately for the development and validation data sets, if applicable.	2-3
	4b	D;V	Specify the key study dates, including start of accrual; end of accrual; and, if applicable, end of follow-up.	2-3
Participants	5a	D;V	Specify key elements of the study setting (e.g., primary care, secondary care, general population) including number and location of centres.	2-3
	5b	D;V	Describe eligibility criteria for participants.	2-3
	5c	D;V	Give details of treatments received, if relevant.	2-3
Outcome	6a	D;V	Clearly define the outcome that is predicted by the prediction model, including how and when assessed.	2-3
	6b	D;V	Report any actions to blind assessment of the outcome to be predicted.	2-3
Predictors	7a	D;V	Clearly define all predictors used in developing or validating the multivariable prediction model, including how and when they were measured.	2-3
	7b	D;V	Report any actions to blind assessment of predictors for the outcome and other predictors.	2-3
Sample size	8	D;V	Explain how the study size was arrived at.	2-3
Missing data	9	D;V	Describe how missing data were handled (e.g., complete-case analysis, single imputation, multiple imputation) with details of any imputation method.	2
Statistical analysis methods	10a	D	Describe how predictors were handled in the analyses.	3
	10b	D	Specify type of model, all model-building procedures (including any predictor selection), and method for internal validation.	3
	10c	V	For validation, describe how the predictions were calculated.	3
	10d	D;V	Specify all measures used to assess model performance and, if relevant, to compare multiple models.	3
	10e	V	Describe any model updating (e.g., recalibration) arising from the validation, if done.	-
Risk groups	11	D;V	Provide details on how risk groups were created, if done.	3
Development vs. validation	12	V	For validation, identify any differences from the development data in setting, eligibility criteria, outcome, and predictors.	4
**Results**				
Participants	13a	D;V	Describe the flow of participants through the study, including the number of participants with and without the outcome and, if applicable, a summary of the follow-up time. A diagram may be helpful.	3
	13b	D;V	Describe the characteristics of the participants (basic demographics, clinical features, available predictors), including the number of participants with missing data for predictors and outcome.	4
	13c	V	For validation, show a comparison with the development data of the distribution of important variables (demographics, predictors and outcome).	-
Model development	14a	D	Specify the number of participants and outcome events in each analysis.	3
	14b	D	If done, report the unadjusted association between each candidate predictor and outcome.	5-6
Model specification	15a	D	Present the full prediction model to allow predictions for individuals (i.e., all regression coefficients, and model intercept or baseline survival at a given time point).	6
	15b	D	Explain how to the use the prediction model.	6-7
Model	16	D;V	Report performance measures (with CIs) for the prediction model.	6-7

performance				
Model-updating	17	V	If done, report the results from any model updating (i.e., model specification, model performance).	6-7
**Discussion**				
Limitations	18	D;V	Discuss any limitations of the study (such as nonrepresentative sample, few events per predictor, missing data).	8
Interpretation	19a	V	For validation, discuss the results with reference to performance in the development data, and any other validation data.	7-8
	19b	D;V	Give an overall interpretation of the results, considering objectives, limitations, results from similar studies, and other relevant evidence.	7-8
Implications	20	D;V	Discuss the potential clinical use of the model and implications for future research.	8
**Other information**				
Supplementary information	21	D;V	Provide information about the availability of supplementary resources, such as study protocol, Web calculator, and data sets.	9
Funding	22	D;V	Give the source of funding and the role of the funders for the present study.	9

Supplementary Table S2. Descriptive characteristics of the participants by sex

Characteristic	Male (n=3128)	Female (n=3909)	P value
Age, years	58.4 (11.2)	58.3 (11.2)	0.853
Height, cm	169.0 (6.1)	156.3 (6.0)	<0.001
Weight, kg	70.0 (10.0)	58.5 (8.9)	<0.001
Body mass index, kg/m^2	24.5 (2.9)	24.0 (3.4)	<0.001
Waist circumference, cm	87.0 (8.1)	80.4 (9.0)	<0.001
Systolic blood pressure, mmHg	122.4 (15.1)	120.3 (17.8)	<0.001
Diastolic blood pressure, mmHg	78.0 (10.4)	74.8 (9.7)	<0.001
Fasting serum glucose, mg/dL	108.2 (27.6)	100.6 (21.0)	<0.001
Total cholesterol, mg/dL	191.3 (39.1)	198.3 (38.7)	<0.001
Triglyceride, mg/dL	165.9 (130.6)	121.5 (76.6)	<0.001
Aspartate aminotransferase, IU/L	25.8 (12.3)	22.6 (10.6)	<0.001
Alanine aminotransferase, IU/L	26.6 (16.7)	19.5 (12.4)	<0.001
Blood urea nitrogen, mg/dL	16.1 (4.5)	15.2 (4.6)	<0.001
Creatinine, mg/dL	1.0 (0.2)	0.7 (0.2)	<0.001
Urinary protein			
Negative*	2552 (81.6)	3513 (89.9)	0.239
Positive**	576 (18.4)	396 (10.1)	
Urinary glucose			
Negative*	2860 (91.4)	3811 (97.5)	0.267
Positive**	268 (8.6)	98 (2.5)	
Urinary pH	5.8 (0.8)	5.9 (0.8)	<0.001
COPD, n (%)			
No	2441 (78.0)	3647 (93.3)	<0.001
Yes	687 (22.0)	262 (6.7)	
Alcohol consumption, n (%)			
No	1140 (36.4)	2872 (73.5)	<0.001
Yes	1988 (63.6)	1037 (26.5)	
Smoking, n (%)			
Never	551 (17.6)	3580 (91.6)	<0.001
Past	1552 (49.6)	170 (4.3)	
Current	1025 (32.8)	159 (4.1)	
Physical activity, n (%)			
No	1882 (60.2)	2465 (63.1)	0.014
Yes	1246 (39.8)	1444 (36.9)	

Data are mean (standard deviation) unless indicated otherwise.

P values calculated using t test (continuous variable) and chi-square test (categorical variable).

Acronyms: COPD, chronic obstructive pulmonary disease.

*negative

**positive (±, +, ++, +++, ++++)

Supplementary Table S3. Univariable analyses of variables involved in the health examination for COPD in male participants

Variable	Estimate	OR (95% CI)	P value	C-index
Age, years	0.090	1.090 (1.080-1.100)	<0.001	0.760
Height, cm	-0.041	0.960 (0.950-0.970)	<0.001	0.573
Weight, kg	-0.046	0.960 (0.950-0.960)	<0.001	0.623
Body mass index, kg/m^2	-0.134	0.870 (0.850-0.900)	<0.001	0.606
Waist circumference, cm	-0.017	0.980 (0.970-0.990)	0.002	0.540
Alcohol consumption, yes (vs. no)	-0.313	0.760 (0.620-0.870)	<0.001	0.537
Smoking			<0.001	0.548
Past vs. Never	0.550	1.730 (1.330-2.260)	<0.001	
Current vs. Never	0.663	1.940 (1.470-2.560)	<0.001	
Physical activity, yes (vs. no)	-0.147	0.860 (0.730-1.030)	0.099	0.517
**Blood pressure**				
Systolic blood pressure, mmHg	0.005	1.005 (0.999-1.011)	0.079	0.528
Diastolic blood pressure, mmHg	-0.057	0.950 (0.940-0.950)	<0.001	0.654
**Diabetes mellitus test**				
Fasting serum glucose, mg/dL	-0.001	0.999 (0.995-1.002)	0.350	0.503
**Dyslipidemia test**				
Total cholesterol, mg/dL	-0.007	0.993 (0.990-0.995)	<0.001	0.583
Triglyceride, mg/dL	-0.002	0.998 (0.997-0.999)	<0.001	0.568
**Liver function test**				
Aspartate aminotransferase, IU/L	-0.008	0.992 (0.984-1.000)	0.048	0.512
Alanine aminotransferase, IU/L	-0.026	0.970 (0.970-0.980)	<0.001	0.602
**Kidney function test**				
Blood urea nitrogen, mg/dl	0.036	1.040 (1.020-1.060)	<0.001	0.543
Creatinine, mg/dl	0.072	1.070 (0.700-1.640)	0.740	0.496
**Urine test**				
Urinary protein	-0.015	0.990 (0.850-1.140)	0.841	0.501
Urinary glucose	-0.091	0.910 (0.820-1.010)	0.076	0.506
Urinary pH	0.039	1.040 (0.940-1.160)	0.466	0.504

OR calculated using logistic regression.

Acronyms: OR, odds ratio; CI, confidence interval.

Supplementary Table S4. Univariable analyses of variables involved in the health examination for COPD in female participants

Variable	Estimate	OR (95% CI)	P value	C-index
Age, years	0.081	1.080 (1.070-1.100)	<0.001	0.742
Height, cm	-0.051	0.950 (0.930-0.970)	<0.001	0.582
Weight, kg	-0.032	0.970 (0.950-0.980)	<0.001	0.563
Body mass index, kg/m^2	-0.034	0.970 (0.930-1.000)	0.077	0.521
Waist circumference, cm	0.009	1.009 (0.995-1.023)	0.207	0.532
Alcohol consumption, yes (vs. no)	-0.352	0.700 (0.520-0.960)	0.021	0.532
Smoking			0.093	0.520
Past vs. Never	0.414	1.510 (0.890-2.580)	0.127	
Current vs. Never	0.488	1.630 (0.960-2.780)	0.073	
Physical activity, yes (vs. no)	-0.346	0.710 (0.540-0.930)	0.012	0.538
**Blood pressure**				
Systolic blood pressure, mmHg	0.014	1.010 (1.010-1.020)	<0.001	0.581
Diastolic blood pressure, mmHg	-0.028	0.970 (0.960-0.990)	<0.001	0.570
**Diabetes mellitus test**				
Fasting serum glucose, mg/dL	0.005	1.005 (1.000-1.010)	0.086	0.531
**Dyslipidemia test**				
Total cholesterol, mg/dL	-0.003	0.997 (0.994-1.000)	0.068	0.532
Triglyceride, mg/dL	0.001	1.001 (1.000-1.003)	0.071	0.563
**Liver function test**				
Aspartate aminotransferase, IU/L	0.004	1.004 (0.994-1.013)	0.459	0.543
Alanine aminotransferase, IU/L	-0.008	0.992 (0.980-1.003)	0.143	0.516
**Kidney function test**				
Blood urea nitrogen, mg/dl	0.057	1.060 (1.030-1.080)	<0.001	0.591
Creatinine, mg/dl	0.721	2.060 (1.240-3.400)	0.002	0.540
**Urine test**				
Urinary protein (vs. Negative)	0.234	1.260 (1.000-1.600)	0.070	0.532
Urinary glucose (vs. Negative)	0.040	1.040 (0.840-1.290)	0.723	0.520
Urinary pH	0.135	1.140 (0.990-1.320)	0.069	0.538

OR calculated using logistic regression.

Acronyms: OR, odds ratio; CI, confidence interval.

Supplementary Table S5. Multivariable model for prediction of COPD when further added smoking as a component

Covariate	Estimate	OR (95% CI)	P value
Intercept	-5.075		<0.001
Age, years	0.094	1.098 (1.089-1.107)	<0.001
Sex, female	-1.032	0.356 (0.279-0.454)	<0.001
Waist circumference, cm	-0.019	0.982 (0.972-0.991)	<0.001
Diastolic blood pressure, mmHg	-0.014	0.986 (0.978-0.995)	0.001
Smoking, past (vs. never)	0.558	1.743 (1.360-2.239)	<0.001
Smoking, current (vs. never)	1.318	3.738 (2.872-4.873)	<0.001

OR calculated using logistic regression.

Acronyms: OR, odds ratio; CI, confidence interval.

Supplementary Table S6. Multivariable model for prediction of COPD in male participants

Covariate	Estimate	OR (95% CI)	P value
Intercept	-2.246		0.003
Age, years	0.085	1.080 (1.070-1.095)	<0.001
Body mass index, kg/m^2	-0.085	0.919 (0.886-0.952)	<0.001
Diastolic blood pressure, mmHg	-0.016	0.984 (0.974-0.994)	0.002
Total cholesterol, mg/dL	-0.001	0.999 (0.996-1.001)	0.317
Triglyceride, mg/dL	0.000	1.000 (1.000-1.001)	0.260
Alanine aminotransferase, IU/L	-0.007	0.992 (0.985-1.000)	0.052
Blood urea nitrogen, mg/dl	-0.019	0.980 (0.960-1.001)	0.064

OR calculated using logistic regression.

Acronyms: OR, odds ratio; CI, confidence interval.

Supplementary Table S7. Multivariable model for prediction of COPD in female participants

**Covariate**	**Estimate**	**OR (95% CI)**	**P value**
Intercept	-14.298		<0.001
Age, years	0.094	1.100 (1.080-1.120)	<0.001
Height, cm	0.051	1.050 (1.020-1.080)	<0.001
Weight, kg	0.033	0.970 (0.950-0.980)	<0.001
Systolic blood pressure, mmHg	-0.003	0.997 (0.989-1.005)	0.420
Blood urea nitrogen, mg/dl	0.005	1.005 (0.978-1.032)	0.718

OR calculated using logistic regression.

Acronyms: OR, odds ratio; CI, confidence interval.
